# Mutation of an L-Type Calcium Channel Gene Leads to T Lymphocyte Dysfunction

**DOI:** 10.3389/fimmu.2019.02473

**Published:** 2019-10-29

**Authors:** Franz Fenninger, Jeffrey Han, Shawna R. Stanwood, Lilian L. Nohara, Hitesh Arora, Kyung Bok Choi, Lonna Munro, Cheryl G. Pfeifer, Iryna Shanina, Marc S. Horwitz, Wilfred A. Jefferies

**Affiliations:** ^1^Michael Smith Laboratories, University of British Columbia, Vancouver, BC, Canada; ^2^Department of Microbiology and Immunology, University of British Columbia, Vancouver, BC, Canada; ^3^Vancouver Prostate Centre, University of British Columbia, Vancouver, BC, Canada; ^4^Centre for Blood Research, University of British Columbia, Vancouver, BC, Canada; ^5^The Djavad Mowafaghian Centre for Brain Health, University of British Columbia, Vancouver, BC, Canada; ^6^Department of Medical Genetics, University of British Columbia, Vancouver, BC, Canada; ^7^Department of Zoology, University of British Columbia, Vancouver, BC, Canada

**Keywords:** *Cacna1f*, L-type calcium channel, Ca_V_1.4, T cell exhaustion, T cell memory, chronic B cell activation, chronic infections, murine gammaherpesvirus 68

## Abstract

Calcium (Ca^2+^) is a vital secondary messenger in T lymphocytes regulating a vast array of important events including maturation, homeostasis, activation, and apoptosis and can enter the cell through CRAC, TRP, and Ca_V_ channels. Here we describe a mutation in the L-type Ca^2+^ channel Ca_V_1.4 leading to T lymphocyte dysfunction, including several hallmarks of immunological exhaustion. Ca_V_1.4-deficient mice exhibited an expansion of central and effector memory T lymphocytes, and an upregulation of inhibitory receptors on several T cell subsets. Moreover, the sustained elevated levels of activation markers on B lymphocytes suggest that they are in a chronic state of activation. Functionally, T lymphocytes exhibited a reduced store-operated Ca^2+^ flux compared to wild-type controls. Finally, modifying environmental conditions by herpes virus infection exacerbated the dysfunctional immune phenotype of the Ca_V_1.4-deficient mice. This is the first example where the mutation of a Ca_V_ channel leads to T lymphocyte dysfunction, including the upregulation of several inhibitory receptors, hallmarks of T cell exhaustion, and establishes the physiological importance of Ca_V_ channel signaling in maintaining a nimble immune system.

## Introduction

Ca^2+^ is a vital signaling molecule in all cells including immune cells and controls important processes like differentiation, homeostasis, activation, proliferation, and apoptosis (1). In lymphocytes, crosslinking the antigen receptor activates a signaling cascade that eventually leads to Ca^2+^ release from the endoplasmic reticulum (ER) into the cytoplasm (2). Upon Ca^2+^ depletion of the ER, Ca^2+^ channels in the plasma membrane open and a Ca^2+^ influx from the extracellular space is triggered. This process is called store-operated Ca^2+^ entry (SOCE) and the main plasma membrane channel involved in it is coined Ca^2+^ release-activated Ca^2+^ (CRAC) channel (3). The CRAC channel consists of the pore-forming unit called ORAI1 and a Ca^2+^-sensing protein named STIM1 that detects low levels of Ca^2+^ in the ER to activate the channel. Loss-of-function mutations in *ORAI1* or *STIM1* genes result in the partial abrogation of SOCE and defective T cell activation (4–7).

Apart from the CRAC channel however, there exist numerous other Ca^2+^ channels in the plasma membrane of lymphocytes that also contribute to the antigen receptor-mediated flux. Among them are the voltage-dependent Ca^2+^ channels (VDCCs), which have emerged as important players in immune cells (8). VDCCs consist of the pore-forming Ca_V_ (α1)-, the β regulatory-, and several other auxiliary subunits. They have been grouped into different families including the L-type Ca^2+^ channels, which are further divided into Ca_V_1.1, 1.2, 1.3, and 1.4. Since they are traditionally activated by a change in membrane potential, these channels have primarily been described in electrically excitable cells but more recent studies have also demonstrated that L-type Ca^2+^ channels play critical roles in murine and human leukocytes (2, 9).

Generally, in acute infections, effector functions of memory CD8 T lymphocytes improve further after a secondary infection. Secondary memory CD8 T lymphocytes are therefore more efficient in fighting pathogens than their primary counterparts. However, in mice it was found that during chronic infections, for example, by lymphocytic choriomeningitis virus (LCMV), secondary memory CD8 T cells were less able to control the infection than primary memory CD8 T lymphocytes. The T lymphocytes exhibit an exhaustion phenotype (10). This is a phenomenon that occurs in many chronic infections where persistent exposure to antigen continuously stimulates T lymphocytes leading to prolonged inflammation. During such conditions, memory T lymphocytes enter an entirely different differentiation program that ends in T cell exhaustion. Exhausted T lymphocytes were first discovered in mice during chronic viral infection in which T lymphocytes became activated but exhibited no effector functions (11). Apart from this lack of effector functions, an exhausted T cell is further characterized by the expression of inhibitory receptors, the inability to survive long-term independent of its cognate antigen, a distinct epigenetic profile and as a result, an altered transcriptome compared to that of effector or memory T lymphocytes.

The inhibitory receptors that exhausted T lymphocytes upregulate include programmed cell death protein 1 (PD-1), lymphocyte activation gene 3 (LAG3), B and T Lymphocyte-Associated protein (BTLA), 2B4, CD160, T cell immunoglobulin domain and mucin domain-containing protein 3 (TIM-3), T cell immunoreceptor with immunoglobulin and ITIM domains (TIGIT) and cytotoxic T lymphocyte-associated protein 4 (CTLA-4) (11, 12). Inhibitory receptors negatively regulate TCR signaling pathways and are usually expressed transiently during activation of T_Eff_ cells to prevent excessive immune responses. Because of their immune-dampening properties, they also play an important role in tolerance and preventing autoimmunity (13). Their sustained expression, however, is typically used to identify exhausted T lymphocytes (11). By targeting inhibitory molecules like PD-1 and CTLA-4 it is possible to modulate the downstream inhibitory pathways and take the brakes off the immune response and reverse exhaustion (14, 15).

Similar as in T lymphocyte exhaustion, L-type Ca^2+^ channel deficiencies often lead to a phenotype that includes impaired TCR signaling, resulting in diminished T cell effector functions and reduced T cell survival (8). Specifically, treatment of Jurkat T lymphocytes with the L-type Ca^2+^ channel inhibitor nifedipine not only lowers TCR-induced Ca^2+^ flux but also results in decreased ERK phosphorylation and subsequent IL-2 production (16). Furthermore, the lack of the common β3 or β4 regulatory subunit of L-type channels gives rise to lessened NFAT translocation and reduced cytokine production in CD4 T lymphocytes (17). CD8 T lymphocytes on the other hand require the β3 subunit for proper cell survival and to prevent lymphocytes from differentiating into memory cells and being chronically activated (18). Our lab was the first to show that the Ca_V_1.4 α1 subunit plays a particularly important role in T cell homeostasis and activation in mice. Specifically, Ca_V_1.4 is required for the survival of naïve T lymphocytes as well as pathogen-specific T cell responses (19). Interestingly, all these phenotypes observed in mice with impaired Ca_V_ channels are also commonly seen during T cell exhaustion.

Here we therefore sought to determine whether Ca_V_1.4 deficiency leads to various exhaustion hallmarks including chronic activation and a memory T cell phenotype as well as study its impact on TCR-induced Ca^2+^ flux. Furthermore, we assessed whether in the absence of Ca_V_1.4 a more serious phenotype may be triggered by an environmental cue provided by murine gamma herpesvirus 68 (MHV-68), a viral ortholog of both Karposi's sarcoma-associated herpesvirus (KSHV) and human Epstein-Barr virus (EBV).

## Materials and Methods

### Mice

Mice were bred at the Animal Research Unit at UBC under specific-pathogen-free conditions. *Cacna1f* –/– mice have been previously described (19, 20) and were backcrossed to C57BL/6NHsd from Harlan Sprague Dawley (Indianapolis, IN, USA) for 13 generations. To eliminate the *Dock2* mutation this colony harbored (21), mice were further backcrossed to C57BL/6NCrl mice from Charles River Laboratories (Wilmington, MA, USA) for one generation. This F1 generation was then allowed to interbreed and in the F2 generation *Cacna1f* –/– only mice were selected to establish a new colony. C57BL/6NCrl mice were also used as control animals. All studies followed ethical guidelines set by both the University of British Columbia's Animal Care Committee and the Canadian Council on Animal Care.

### Splenocyte Isolation

8 to 14-week-old mice were euthanized and their spleens excised. Spleens were then mashed through a 70 μm cell strainer using a syringe plunger to obtain a single cell suspension. Red blood cells were then lysed using ACK lysing buffer and the remaining white blood cells were washed with PBS and resuspended in PBS with 2% FBS.

### Flow Cytometry Surface Staining

2 × 10^6^ freshly isolated mouse splenocytes, resuspended in PBS with 2% FBS, were labeled for 30 min at 4°C in the dark with different antibody panels. The antibodies used were: α-CD8 (5H10, Thermo Fisher, cat. nr. MCD0828TR), α-CD4 (RM4-5, Thermo Fisher, cat. nr. 56-0042-80), α-CD3 (17A2, eBioscience, cat. nr. 46-0032-80), α-CD62L (MEL-14, Biolegend, cat. nr. 104411), α-CD44 (IM7, Biolegend, cat. nr. 103005), α-PD-1 (29F-1A12, Biolegend, cat. nr. 135223), α-IL7R (A7R34, Biolegend, cat. nr. 135031), α-CTLA-4 (UC10-4B9, eBioscience, cat. nr. 12-1522-81), α-B220 (RA3-6B2, eBioscience, cat. nr. 47-0452-82), α-CD21 (eBio4E3, eBioscience, cat. nr. 48-0212-80), α-CD23 (B3B4, Biolegend, cat. nr. 101612), α-CD86 (GL1, eBioscience, cat. nr. 12-0862-81), α-CD69 (H1.2F3, eBioscience, cat. nr. 11-0691-81), α-IAb (AF6-120.1, eBioscience, cat. nr. 46-5320-80). The cells were then washed twice with PBS, resuspended in PBS with 2% FBS and data were acquired on an LSRII Flow Cytometer (BD Biosciences) and analyzed with FlowJo software, ver. 9.9.6 (Treestar, Inc).

### Ca^2+^ Flux Assay

4 × 10^6^ freshly isolated mouse splenocytes, resuspended in HBSS with 2% FBS, were labeled with 1 μM Fluo-4 and 2 μM Fura Red (Thermo Fisher, cat. nr. F14201, F3021) for 45 min at room temperature in the dark. Cells were then washed, stained with α-B220 (RA3-6B2, eBioscience, cat. nr. 47-0452-82), α-CD8 (5H10, Thermo Fisher, cat. nr. MCD0828TR), and α-CD4 (RM4-5, Thermo Fisher, cat. nr. 56-0042-80) for 30 min on ice, washed again and resuspended in HBSS with 2% FBS. After prewarming the cells for 15 min at 37°C, baseline Ca^2+^ levels were acquired for 30s. 1 μM of thapsigargin (Thermo Fisher, cat. nr. T7458) was then added to the cells and acquisition was continued for a total of 3 min. Data were acquired on an LSRII (BD Biosciences) and analyzed with FlowJo software (Treestar, Inc).

### MHV-68 Infection

Eight-week-old mice were injected intraperitoneally with 10^4^ pfus of MHV-68 WUMS strain (purchased from ATCC cat. nt. VR-1465, propagated on BHK cells) and sacrificed 4 weeks later. Their spleens were then harvested and processed for flow cytometry analysis.

### Statistical Tests

Statistical significance was, if not indicated otherwise, determined using unpaired Student's *t-*tests using RStudio. For the MHV-68 infection experiment interaction of genotype and infection status was determined using two-way ANOVA. For some mouse experiments, data of 3–4 different experiments, each using 3–5 animals per experimental group, were pooled. In that case the experiments were performed using the same flow cytometry antibodies (same vials) and the exact same laser settings on the flow cytometer.

## Results

### *Cacna1f* Gene Is Disrupted

**Figure 1:**
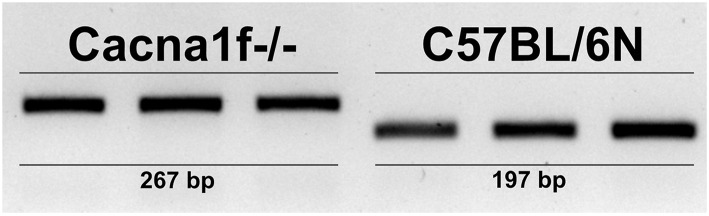
Ca_V_1.4-deficient mice have an insert in their *Cacna1f* gene. Genotyping of three KO (*Cacna1f*–/–) and WT (C57BL/6N) demonstrates that KO mice carry a 70 bp insert in exon 7 of their *Cacna1f* gene.

**Figure 2:**
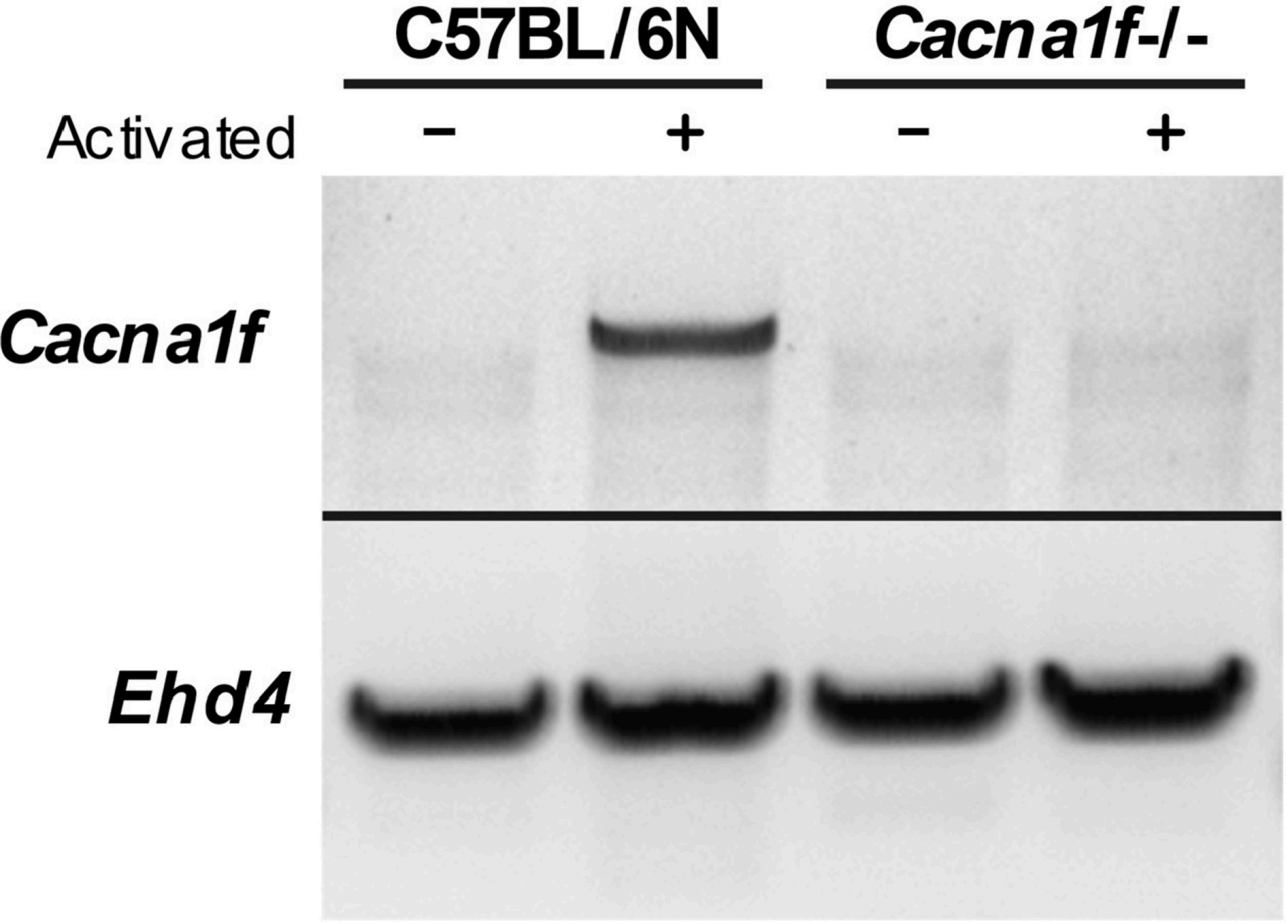
Ca_V_1.4-deficient mice do not express *Cacna1f* mRNA upon activation. RNA from unstimulated and α-CD3 and α-CD28 stimulated murine naïve T lymphocytes was converted to cDNA. The cDNA was used as a template to amplify *Cacna1f* transcripts (4327 bp) by RT-PCR. *Ehd4* (2331 bp) was used as a control gene.

The *Cacna1f* gene of our mouse model is disrupted by an insert in exon 7, leading to a premature stop codon (20). We first verified the presence of this insert in the *Cacna1f* –/– mouse model (Figure 1). Additionally, we have shown that Ca_V_1.4 KO mice do not express *Cacna1f* mRNA upon activation (Figure 2).

### Ca_V_1.4-Deficient Mice Exhibit a Reduced Frequency of CD8 T Lymphocytes

**Figure 3:**
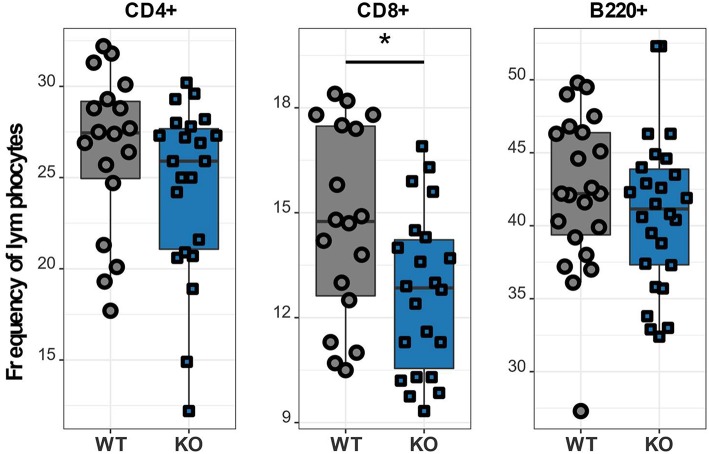
*Cacna1f*–/– mice have a reduced frequency of splenic CD8 T lymphocytes. Splenocytes of WT (*n* = 18 or *n* = 22) and KO (*n* = 22 or *n* = 26) mice were stained with different antibodies and analyzed by flow cytometry. Boxplots show four pooled experiments. *p < 0.05.

After verifying the disruption of the *Cacna1f* gene we investigated details of the T lymphocyte phenotype affected by the Ca_V_1.4 deficiency. To further characterize the immune phenotype of this Ca_V_1.4 KO mouse model, we harvested spleens of WT and *Cacna1f*–/– mice and used a variety of flow cytometry-based assays to analyse their splenocytes. We initially examined the abundance of CD4 and CD8 T lymphocytes as well as B lymphocytes in these mice and found that Ca_V_1.4 deficiency in mice led to a modest reduction of CD8 T lymphocyte frequency. The frequencies of CD4 T lymphocytes and of B lymphocytes were similar to those of WT mice (Figure 3).

### Ca_V_1.4 Deficiency Leads to a Memory T Cell Phenotype

**Figure 4:**
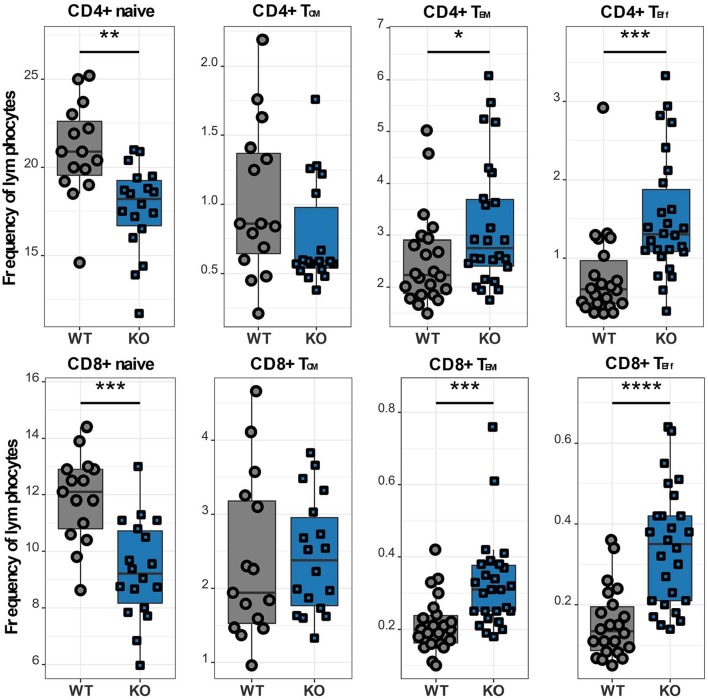
Ca_V_1.4 deficiency results in a memory T cell phenotype. Splenocytes of WT (*n* = 15 or *n* = 22) and KO (*n* = 18 or *n* = 26) mice were stained with antibodies against CD3, CD4, CD8, CD62L, IL7R, and CD44 and analyzed by flow cytometry. The different population frequencies shown in boxplots are classified as CD62L+ IL-7R+ CD44- (naïve), CD62L+ IL-7R+ CD44+ (T_CM_), CD62L- IL-7R+ (T_EM_), CD62L- IL-7R- (end-stage T_Eff_). Boxplots show four pooled experiments *p < 0.05, **p < 0.01, ***p < 0.001, ****p < 0.0001.

In order to further investigate different T cell subpopulations, we next examined the distribution of T lymphocytes into naïve, memory and effector cell subsets by flow cytometry. In murine T cells, IL7R is downregulated upon activation and re-expressed on memory T lymphocytes, which can be further divided into two subsets: effector memory T lymphocytes (T_EM_), which are CD62L- CCR7-, and CD44+ and central memory T lymphocytes (T_CM_), which are CD62L+ CCR7+ and CD44+ (12, 22). We classified CD62L+ IL7R+ CD44- as naïve, CD62L+ IL7R+ CD44+ as T_CM_, CD62L- IL7R+ as T_EM_ and CD62L- IL7R- as T_Eff_ cells (Supplementary Figure 1). Ca_V_1.4 KO mice analyzed using this gating strategy exhibited a memory T cell phenotype, i.e. although T_CM_ cell frequencies of Ca_V_1.4 KO mice were comparable to WT levels, their CD4 and CD8 T_EM_ subsets were significantly increased. Also, the *Cacna1f*-/- mice had an increased frequency of T_Eff_ cells, again for CD4 and CD8 T lymphocytes. Accordingly, the naïve T cell frequency was reduced (Figure 4).

### Ca_V_1.4-Deficient T Lymphocytes Are Continuously Activated and Potentially Exhausted

**Figure 5:**
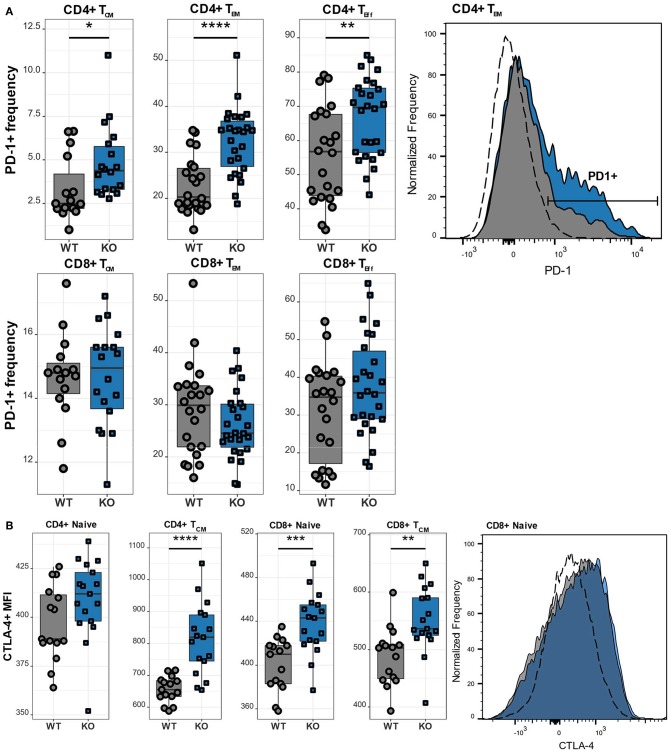
Ca_V_1.4 deficiency results in T cell activation/potential exhaustion. Splenocytes of WT (*n* = 15 or *n* = 22) and KO (*n* = 18 or *n* = 26) mice were stained with different antibodies and analyzed by flow cytometry. **(A)** The PD-1+ frequency and **(B)** CTLA-4 mean fluorescent intensity (MFI) are shown in boxplots for different populations, which are classified as CD62L+ IL-7R+ CD44- (naïve), CD62L+ IL-7R+ CD44+ (T_CM_), CD62L- IL-7R+ (T_EM_), CD62L- IL-7R- (end-stage T_Eff_). Boxplots show four pooled experiments. Histograms show one representative sample of each genotype. The dashed lines show FMO controls. *p < 0.05, **p < 0.01, ***p < 0.001, ****p < 0.0001, MFI = mean fluorescent intensity.

Upon antigen recognition, T lymphocytes upregulate various activation and inhibitory markers, including PD-1 and CTLA-4. Once the antigen has been cleared, the T lymphocytes downregulate the expression of these surface markers. However, if the T lymphocytes are repeatedly challenged as it happens in chronic infections, they become exhausted and the continuous upregulation of the aforementioned surface markers is a hallmark of exhaustion. We therefore next examined the expression of the inhibitory receptors PD-1 and CTLA-4 in different T cell subsets. CD4 T lymphocytes exhibited high expression of PD-1 (Figure 5A), which was particularly pronounced in T_CM_, T_EM_, as well as T_Eff_ cells. Interestingly, this increase of PD-1 was restricted to the CD4 subset as CD8 T lymphocytes had normal levels of PD-1. While PD-1 is known to be upregulated on antigen-experienced T lymphocytes, CTLA-4 is functional during the initial phase of T cell activation and is therefore found on naïve T lymphocytes (23). Accordingly, we observed increased CTLA-4 levels in Ca_V_1.4 KO naïve T lymphocytes, particularly in the CD8 subset. In addition, T_CM_ cells of both CD4 and CD8 subsets exhibited increased expression of the inhibitory marker (Figure 5B).

### Ca_V_1.4 Deficiency Causes Chronic B Lymophocyte Activation

**Figure 6:**
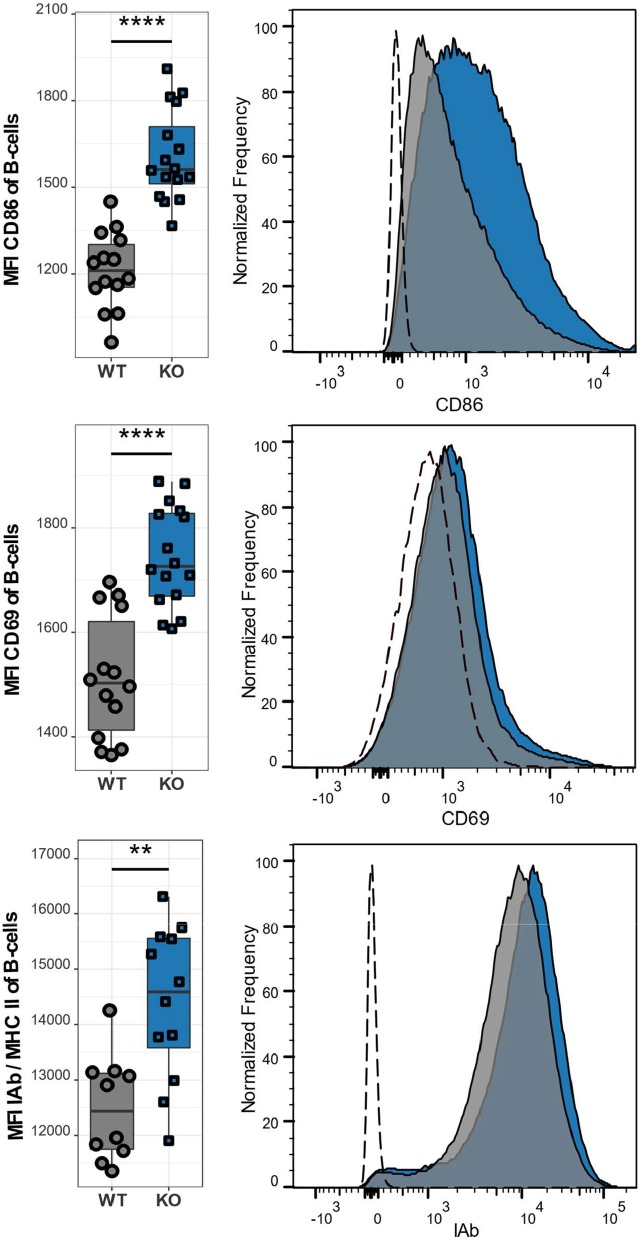
Ca_V_1.4 deficiency leads to an activated B cell phenotype. Splenocytes of WT (*n* = 14 or *n* = 10) and KO (*n* = 16 or *n* = 12) mice were stained with antibodies against B220, CD86, CD69, and IAb and analyzed by flow cytometry. The population quantified consists of B lymphocytes only (B220+). Boxplots show four pooled experiments. Histograms show one representative sample of each genotype. The dashed lines show FMO controls. ^**^p < 0.01, ****p < 0.0001, MFI = mean fluorescent intensity.

Besides the abnormal T cell phenotype, we also examined the effect of Ca_V_1.4 deficiency on B lymphocytes. Similar to T lymphocytes, B lymphocytes of *Cacna1f*–/– mice are in an activated state as demonstrated by the high expression levels of the activation markers CD86, CD69, and MHCII (Figure 6). Our observations were consistent, regardless of the age of the mice, which ranged from 8 to 14 weeks. This suggests that the memory T cell phenotype and upregulation of inhibitory receptors, as well as the activation status of B lymphocytes are chronic conditions.

### Ca_V_1.4-Deficient T but Not B Lymphocytes Exhibit a Reduced Ca^2+^ Flux

**Figure 7:**
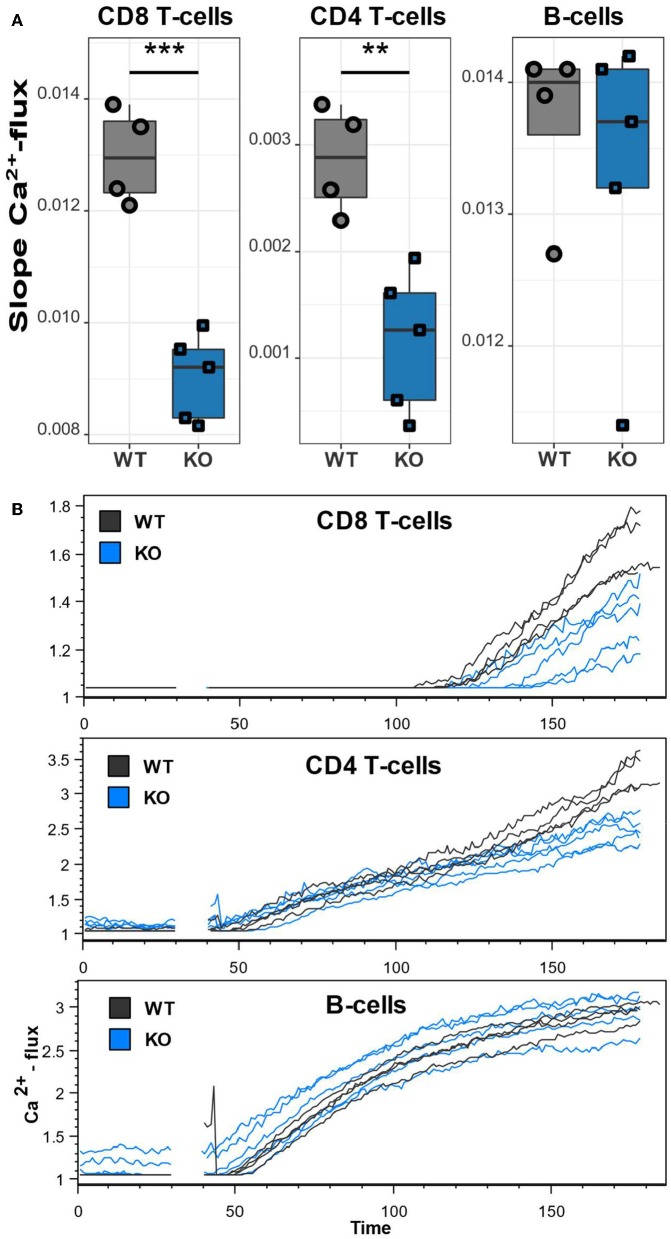
Ca_V_1.4-deficient T lymphocytes exhibit a reduced Ca^2+^ flux. Splenocytes of WT (*n* = 4) and KO (*n* = 5) mice were stained with Ca^2+^ dyes and different T and B cell-specific antibodies and analyzed by flow cytometry. Thapsigargin was added to cells after 30 s of acquisition. **(A)** The boxplots show the quantified slopes of increasing Ca^2+^ concentration for each cell type. **(B)** The flow cytometry kinetics plots show the actual Ca^2+^ influx over time. Representative of two independent experiments. ^**^p < 0.01, ***p < 0.001.

The unusually high frequencies of memory T lymphocyte subsets and upregulation of inhibitory receptors/activation markers on T lymphocytes and B lymphocytes indicate a lymphocyte dysfunction as a result of the mutated *Cacna1f* gene. To assess the functional impact of Ca_V_1.4 deficiency, we next measured the Ca^2+^ flux in splenic CD4 and CD8 T lymphocytes as well as B lymphocytes, after thapsigargin stimulation. Thapsigargin triggers a flux of extracellular Ca^2+^ into the cytoplasm by blocking the reuptake of Ca^2+^ into the ER. This leads to SOCE without engaging the antigen receptor and triggering the associated TCR/BCR signaling pathway. While B lymphocytes of Ca_V_1.4 KO mice had an unchanged Ca^2+^ flux compared to WT lymphocytes, CD8 and CD4 T lymphocytes both showed a significantly reduced flux after stimulation (Figure 7). These data suggest that Ca_V_1.4 is also involved during SOCE in T lymphocytes and that its absence leads to a reduction of the induced Ca^2+^ flux.

### Ca_V_1.4 KO Mice Exhibit an Increased CD4/CD8 T Lymphocyte Ratio Post-infection With MHV-68

**Figure 8:**
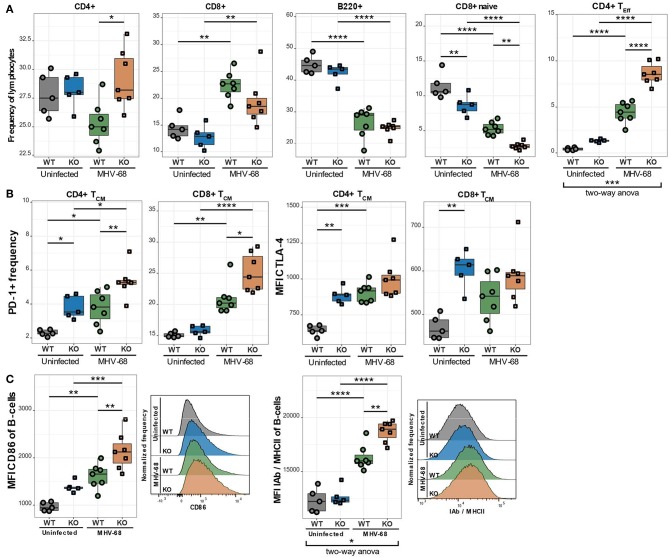
MHV-68-infected Ca_V_1.4 KO mice have fewer CD8+ naïve but more CD4+ T_Eff_ cells and exhibit further upregulation of exhaustion markers on T_CM_ cells as well as activation markers on B lymphocytes. Splenocytes of uninfected (*n* = 5) and MHV-68-infected (*n* = 7) WT and KO mice were stained with different antibodies and analyzed by flow cytometry. **(A)** The different population frequencies shown in boxplots are classified as CD62L+ IL-7R+ CD44- (naïve T lymphocytes), CD62L+ IL-7R+ CD44+ (T_CM_; central memory T lymphocytes), CD62L- IL-7R- (end-stage T_Eff_; effector T lymphocytes). **(B)** PD-1 frequency and CTLA-4 MFI were quantified in T_CM_ cells. **(C)** Activation markers (CD86 and IAb/MHCII) were assessed in B lymphocytes (CD19+). Histograms show one representative sample of each experimental group. Experiment was only performed once. *p < 0.05, **p < 0.01, ***p < 0.001, ****p < 0.0001. MFI = mean fluorescent intensity.

As already discussed in the general introduction, environmental factors can significantly alter the progression of various diseases. In many PIDs, like XLP syndrome for example, environmental cues, such as an EBV infection, trigger or exacerbate the disease. We therefore postulated that EBV infection could also aggravate the observed immune phenotype in Ca_V_1.4 deficiency. To investigate this hypothesis, we infected WT and *Cacna1f*–/– mice with murine gamma herpesvirus 68 (MHV-68), which is utilized to model human EBV infections as the disease progression is very similar (24). We then sacrificed the animals 4 weeks post-infection and analyzed their splenic lymphocyte populations. Interestingly, the Ca_V_1.4 KO animals had an increased CD4/CD8 T cell ratio compared to WT animals, post-infection, which was mostly due to a higher frequency of CD4 T lymphocytes. The B lymphocyte frequencies were reduced equally in WT and Ca_V_1.4 KO animals (Figure 8A).

### Ca_V_1.4 Deficiency Leads to a Higher CD4 T_Eff_ Lymphocyte Frequency Post-infection

To characterize the T cell subpopulations in more detail we analyzed the frequencies of naïve, memory and effector T lymphocytes post-infection. Ca_V_1.4 KO animals exhibited a significantly higher CD4 T_Eff_ cell frequency than WT mice. This change was particularly prominent as it demonstrated a significant interaction between the genotype (WT or KO) and infection status (uninfected or infected) of the mice according to a two-way ANOVA (Figure 8A). The frequency of naïve CD8 T lymphocytes was reduced in WT and KO animals equally (Figure 8A). Interestingly, in KO mice, the frequency of CD8 T_CM_ cells was also reduced upon infection while the WT levels remained unchanged (not shown). CD4 T_CM_ cell frequencies, on the other hand, were diminished similarly in WT and KO mice post-infection. The T_EM_ cell frequencies of both WT and KO animals increased to equal levels post-infection (not shown). Overall, the increased production of CD4 T_Eff_ lymphocytes in Ca_V_1.4-deficient mice was the most striking T cell subpopulation change compared to WT animals after MHV-68 infection.

### Ca_V_1.4-Deficient T_CM_ Cells Upregulate Inhibitory Receptors Post-infection

When analyzing IRs post MHV-68 infection, we found that the PD-1+ frequency increased in the T_CM_ subset for WT and KO animals but was still significantly higher in Ca_V_1.4 KO T lymphocytes compared to WT lymphocytes (Figure 8B). CTLA-4 expression increased only on WT T_CM_ cells post-infection so that it became comparable to that of Ca_V_1.4 KO cells. None of the expression changes in IRs, however, exhibited a statistically significant interaction between genotype and infection status of the mice.

### Chronic B Lymphocyte Activation Is Amplified in Ca_V_1.4 KO Mice Post-infection

The examined B cell activation markers were all further upregulated after MHV-68 infection in both WT and KO mice but were significantly more pronounced in the KO animals (Figure 8C). Specifically, the expression of MHC-II was dependent on the interaction of genotype and infection status according to a two-way ANOVA. Overall, the MHV-68 infection increased the memory T cell frequency and upregulation of inhibitory receptors similarly in WT and KO mice, while the chronic B cell activation (specifically increased MHC-II expression) and the expansion of CD4 T_Eff_ cells were significantly more pronounced in the Ca_V_1.4 KO animals.

## Discussion

Although CRAC channels are known to be the main route of entry of Ca^2+^ into T lymphocytes, there also exists an array of other channels that contribute to TCR-mediated Ca^2+^ flux. Among them are the L-type voltage-gated Ca^2+^ channels, including Ca_V_1.4 whose role in T cell homeostasis and activation has been described previously by our lab (19). Here we further investigate the phenotype of the mouse model and find that Ca_V_1.4 deficiency leads to an activated memory T cell phenotype. This is reflected in the decreased frequency of naïve T lymphocytes, and increase of T_Eff_ and T_EM_ lymphocyte frequencies in CD4 and CD8 subsets. A memory T cell phenotype based on CD44 expression has already been described previously by our lab (19) as well as by Jha et al. in a KO mouse model of the β3 regulatory subunit that also constitutes the Ca_V_1.4 channel (18). While the frequency of total CD4 T lymphocytes did not change, the CD8 T lymphocyte subset is modestly reduced in frequency in *Cacna1f*–/– mice, which is also consistent with the β3 KO mouse of Jha et al. (18).

The upregulation of CTLA-4 on naïve and T_CM_ cells is a novel phenotype of Ca_V_1.4 KO mice that has not been reported previously. CTLA-4 is a homolog of CD28, a co-receptor on the surface of T lymphocytes. It competes for the binding of the ligand B7 on APCs but unlike CD28 it does not produce a stimulatory signal but instead inhibits T cell activation (23). This could potentially explain the reduced TCR-mediated proliferation capacity observed previously in *Cacna1f* and β3-deficient mice (18, 19). Also, treatment of mice with nifedipine, an L-type Ca^2+^ channel antagonist, was shown to inhibit the proliferation of splenic T lymphocytes, indicating that Ca_V_1.4 might be essential for T lymphocytes to divide (16).

PD-1 is another inhibitory marker that can interfere with phosphorylation in TCR signaling and as a result, reduce proliferation and effector functions. Also, this marker was upregulated in *Cacna1f*–/– mice, which has already been described previously in our lab (19). Here we show that this PD-1 upregulation is confined to CD4 T_CM_, T_EM_ and T_Eff_ cells. Also, the B lymphocytes of *Cacna1f*–/– mice exhibit an activated phenotype as seen by the upregulation of the activation markers CD86, CD69, and MHC-II. During T-dependent antigen responses, B lymphocytes require help from CD4 T lymphocytes to be activated. CD4 T_FH_ lymphocytes are a specialized subset of T lymphocytes for this function and provide co-stimulatory signals via expression / secretion of CD40L, IL-21, and IL-4 (25). Possibly, the activated phenotype of CD4 T lymphocytes also leads to an increase of these stimulatory signals that activate B lymphocytes. In this context it is interesting to note that PD-1 is typically found on T_FH_ lymphocytes (26). The increase of PD-1 on CD4 T lymphocytes could also be the result of the expansion of the T_FH_ lymphocyte subset. An increase of this population could potentially also cause the chronic B cell activation. Interestingly, we only observed a reduced Ca^2+^ flux in Ca_V_1.4 KO T lymphocytes but not in Ca_V_1.4 KO B lymphocytes. This also supports the hypothesis that Ca_V_1.4 deficiency has a T lymphocyte intrinsic effect and the impact on B lymphocytes is of secondary nature. Further investigation will be required to confirm this hypothesis.

The reduced Ca^2+^ mobilization in T lymphocytes was also seen in previous work in our lab with this mouse model (19). In β3 KO CD4 T lymphocytes, CD3 crosslinking also led to a diminished Ca^2+^ flux but, conversely, thapsigargin-induced Ca^2+^ flux was normal (17). However, both KO mouse models displayed impaired nuclear translocation of NFAT, which has been shown to result in reduced cytokine production (17–19). In our α1 subunit KO mouse, it also led to impaired proliferation responses (19).

Our results demonstrate that Ca_V_1.4 deficiency leads to a chronically activated phenotype of T and B lymphocytes as it is often seen in T lymphocyte exhaustion during chronic infections. Since the mouse facility where these animals were housed is free of pathogens, it is likely that the phenotype arose without an infectious trigger. However, in many genetic PIDs, for example XLP syndrome, the disease is only triggered after an EBV infection. To establish whether these kinds of environmental factors also play a role in Ca_V_1.4 deficiency, we infected WT and KO mice with MHV-68, a murine model virus for EBV. The infection model revealed that the phenotypes are amplified in Ca_V_1.4 KO mice during MHV-68 infection. Specifically, the frequency of CD4 T_Eff_ cells was disproportionately increased in *Cacna1f*–/– mice 4 weeks post-infection and demonstrates that Ca_V_1.4 deficiency leads to a skewed T lymphocyte response. It is possible that due to impaired effector function there is a need to produce more T_Eff_ lymphocytes in order to control MHV-68 infection in Ca_V_1.4 KO mice. This is consistent with the observations that it is mostly CD4 T lymphocytes that control an MHV-68 infection (27). Furthermore, the higher expression of PD-1 seen on CD4 but not CD8 subsets before infection might provide a stronger inhibitory signal that dampens CD4 T cell responses and necessitates a higher number of CD4 T_Eff_ cells.

The inhibitory marker CTLA-4 and particularly PD-1 were further upregulated in T_CM_ cells after MHV-68 infection. Since T_FH_ cells are known to express PD-1, the upregulation of the marker could also mean an increase of the T_FH_ population. During primary infection, MHV-68 targets alveolar cells and splenocytes and then goes dormant in splenic B lymphocytes, causing a chronic infection. Interestingly, this B cell latency requires the help of T_FH_ cells (24). Thus, the significant increase of activation markers (particularly MHCII) on Ca_V_1.4-deficient B lymphocytes after MHV-68 infection could be a result of an increased T_FH_ population providing more help for MHV-68 to infect B lymphocytes in Ca_V_1.4 KO mice than in WT. Given the increased frequency of CD4 T_Eff_ lymphocytes and augmented activation status of B lymphocytes it would be of interest to examine the viral load in Ca_V_1.4-deficient B lymphocytes in the future.

The MHV-68 infection model demonstrates that the immune phenotype of the Ca_V_1.4 KO mice can be aggravated by environmental factors. It is possible that other bacterial/viral infections of pathogenic or commensal nature also have similar effects. Since the housing conditions are pathogen-free, this often provides a setting in which immunodeficient mice can thrive as they do not encounter the same ecological challenges as they would in the wild.

While the expression of inhibitory receptors, as well as diminished Ca^2+^ flux, suggests the exhaustion of T lymphocytes in Ca_V_1.4-deficient mice, definitive proof will have to be provided by assessing T lymphocyte functions, such as cytotoxicity. Additionally, other infection models (e.g., LCMV) will provide further insights into the T cell dysfunction caused by Ca_V_1.4 deficiency and how it interacts with environmental factors.

## Data Availability Statement

The raw data supporting the conclusions of this manuscript will be made available by the authors, without undue reservation, to any qualified researcher.

## Ethics Statement

The study's protocol was approved by both the University of British Columbia's Animal Care Committee and the Canadian Council on Animal Care.

## Author Contributions

FF, MH, and WJ: designed research. FF, SS, JH, LN, HA, KC, LM, and IS: performed research. FF and WJ: analyzed data. FF, SS, KC, LM, CP, MH, and WJ: edited paper. FF and WJ: wrote paper.

### Conflict of Interest

WJ declares funding from Pascal Biosciences Inc. for studying L-type Ca^2+^ channels unrelated to this study. The remaining authors declare that the research was conducted in the absence of any commercial or financial relationships that could be construed as a potential conflict of interest.
