# Supplementary material for: Mutation of an L-Type Calcium Channel Gene Leads to T Lymphocyte Dysfunction
Source: Front Immunol. 2019 Oct 29;10:2473. doi: 10.3389/fimmu.2019.02473 (PMC6833481; doi:10.3389/fimmu.2019.02473)
Supplement: Supplementary file 1 [file Image_1.pdf]

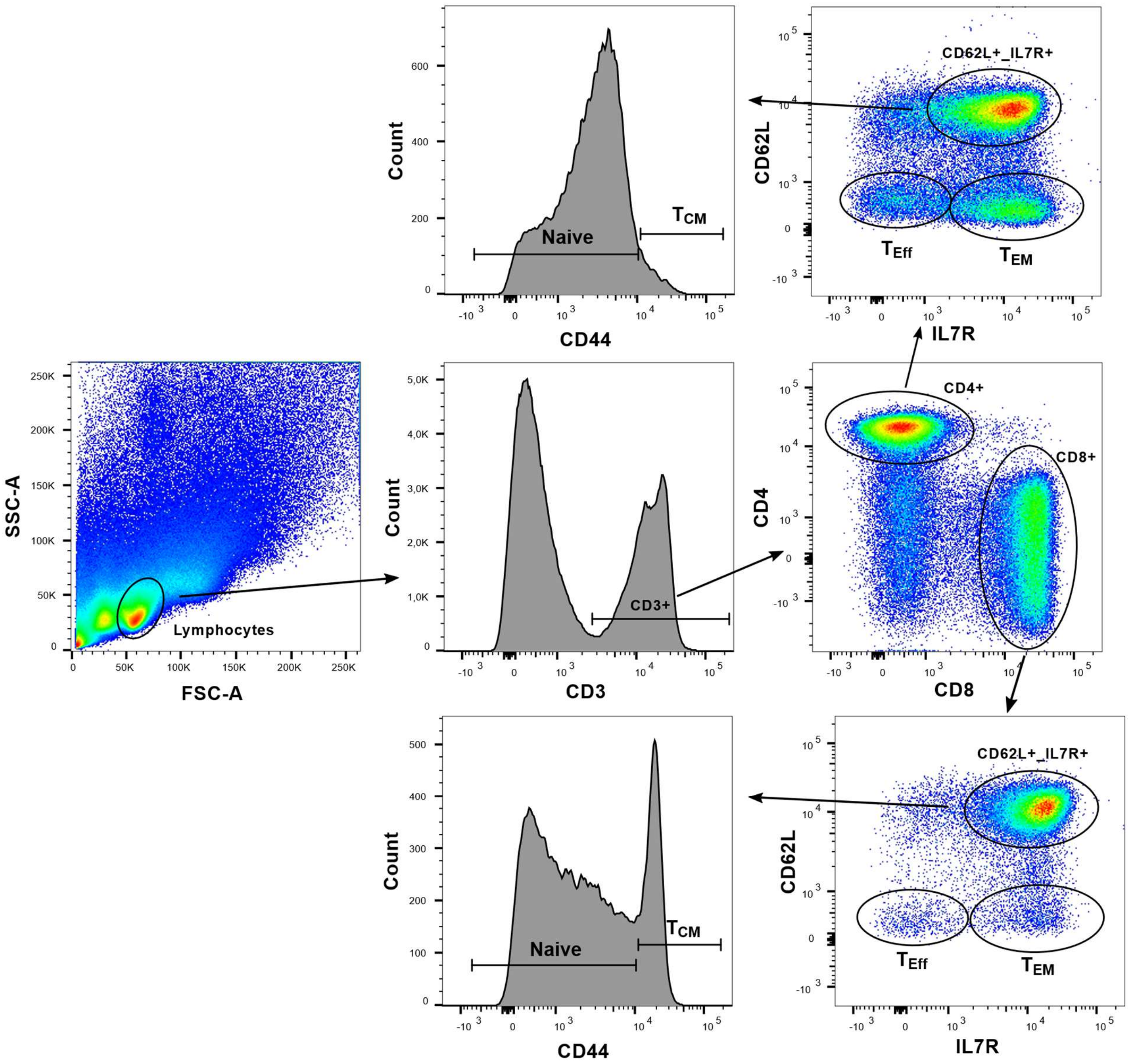

Supplementary Figure 1. Gating strategy for mouse splenocytes

The different populations were classified as CD62L+ IL-7R+ CD44- (naïve), CD62L+ IL-7R+ CD44+ (T_{CM}), CD62L- IL-7R+ (T_{EM}), CD62L- IL-7R- (end-stage T_{Eff}).
